# *Pax6* in Collembola: Adaptive Evolution of Eye Regression

**DOI:** 10.1038/srep20800

**Published:** 2016-02-09

**Authors:** Ya-Nan Hou, Sheng Li, Yun-Xia Luan

**Affiliations:** 1 Key Laboratory of Insect Developmental and Evolutionary Biology, Institute of Plant Physiology & Ecology, Shanghai Institutes for Biological Sciences, Chinese Academy of Sciences, Shanghai, China

## Abstract

Unlike the compound eyes in insects, collembolan eyes are comparatively simple: some species have eyes with different numbers of ocelli (1 + 1 to 8 + 8), and some species have no apparent eye structures. P*ax6* is a universal master control gene for eye morphogenesis. In this study, full-length *Pax6* cDNAs, *Fc-Pax6* and *Cd-Pax6*, were cloned from an eyeless collembolan (*Folsomia candida*, soil-dwelling) and an eyed one (*Ceratophysella denticulata*, surface-dwelling), respectively. Their phylogenetic positions are between the two *Pax6* paralogs in insects, *eyeless* (*ey*) and *twin of eyeless* (*toy*), and their protein sequences are more similar to Ey than to Toy. Both Fc-Pax6 and Cd-Pax6 could induce ectopic eyes in *Drosophila*, while Fc-Pax6 exhibited much weaker transactivation ability than Cd-Pax6. The C-terminus of collembolan Pax6 is indispensable for its transactivation ability, and determines the differences of transactivation ability between Fc-Pax6 and Cd-Pax6. One of the possible reasons is that *Fc-Pax6* accumulated more mutations at some key functional sites of C-terminus under a lower selection pressure on eye development due to the dark habitats of *F. candida*. The composite data provide a first molecular evidence for the monophyletic origin of collembolan eyes, and indicate the eye degeneration of collembolans is caused by adaptive evolution.

Because of the structural and functional differences among various types of animal eyes, eye evolution is a difficult, yet fascinating mystery to explore. In 1872, Darwin proposed that all complex eyes of animals evolved from a simple prototypic eye that consisted of a photoreceptor cell and a pigment cell^1^. However, Neo-Darwinists assume that the eye evolved independently 40–60 times in various taxa^2^. The discovery of *Pax6* as a universal master control gene for eye development throughout the animal kingdom supports the hypothesis of the monophyletic origin of different eye types^3^,^4^.

As an evolutionarily conserved gene in all bilaterian animals, the typical *Pax6* isolated to date encode proteins that include two highly conserved DNA-binding domains–a paired domain (PD, 128 amino acids) and a paired-like homeodomain (HD, 60 amino acids), along with a short N-terminus (NT), a glycine-rich central linker region (B) that connects the PD and HD, and a flexible proline, serine and threonine (PST)-rich C-terminal tail (CT)^5^.

*Pax6* was initially cloned from humans^6^, mice^7^, and zebrafish^8^. Two *Pax6* paralogs–*eyeless* (*ey*) and *twin of eyeless* (*toy*)–have been identified in some higher insects: *Drosophila melanogaster*^9^,^10^, *Apis mellifera*^11^,^12^, *Tribolium castaneum*^13^, and *Nasonia vitripennis*^14^,^15^. *Toy* and *ey* share similar expression patterns in the developing visual system, but Toy is more similar to vertebrate Pax6 in terms of the size, the C-terminal sequences, DNA-binding function, protein structure, and early embryonic expression patterns. Toy acts upstream of Ey in *Drosophila*^10^, and both Toy and Ey can bind to common or different downstream targets^16^.

Loss-of-function mutations in *Pax6* result in small eyes in mice^17^ and rats^18^, aniridia in humans^19^,^20^,^21^, eyeless or headless phenotype in *Drosophila*^9^,^22^, or gross abnormalities in head morphogenesis in *C. elegans*^23^. Misexpression of *Pax6* homologs of mouse^3^, *Drosophila*^3^, sea squirt^24^, squid^25^, and lancelet^26^ in *Drosophila* by means of the GAL4/UAS system can induce ectopic *Drosophila*-type compound eyes. Misexpression of *Pax6* in *Xenopus* embryos can result in small but fully differentiated ectopic *Xenopus* lens structures^27^. In addition, ectopic vertebrate eye structures can also be induced by expressing the *Drosophila ey* or *toy* genes in *Xenopus* embryos^28^. These studies suggest that the highly conserved *Pax6* controls the development of various types of eyes in both invertebrates and vertebrates. However, to date, functional studies of Pax6 in relatively primitive hexapods are few, especially for comparative studies of closely-related species with distinct eye phenotypes.

Among the smallest yet most diverse hexapods in the world, collembolans are found in large numbers in almost all habitats^29^. Uniquely, different from the compound eyes of most arthropod species, collembolans have no eyes or have only simple ocelli on each side of the head with different numbers of ocelli (0 + 0, 1 + 1, 2 + 2, 3 + 3, 5 + 5, 6 + 6, or 8 + 8)^29^. Scanning electron microscope (SEM) analysis suggests that the location of collembolan eye fields is similar to that of insects^30^,^31^, and the structure of collembolan ocellus resembles that of the ommatidium from insects and crustaceans^32^,^33^. The behavioral assays and SEM analyses showed the eyeless species made the same phototactic choices as the eyed species, and the eyeless species may have internal photoreceptors or other photosensitive structures and retained the ability to detect and distinguish ultraviolet (UV), white light and darkness^34^. To date, no molecular studies regarding the genetic regulation and evolution of collembolan eyes have been performed.

In this study, we cloned and compared the full-length *Pax6* coding sequences: *Fc-Pax6* from the eyeless soil-dwelling collembolan (*Folsomia candida*, Fig. 1A,A’) and *Cd-Pax6* from the surface-dwelling collembolan (*Ceratophysella denticulata*, Fig. 1B) bearing 8 + 8 ocelli (Fig. 1B’). The phylogenetic analysis of *Pax6* in Hexapoda suggest *Fc-Pax6* and *Cd-Pax6* form an independent clade, which is closer to insect *ey* than to insect *toy*. Misexpression of these two collembolan *Pax6* genes in *Drosophila* determined that Fc-Pax6 exhibited much weaker transactivation ability than Cd-Pax6. This study of collembolan *Pax6* is a good case of adaptive evolution of eye regression.

## Results

### The collembolan Pax6 is more similar to insect Ey than to insect Toy

**Figure 1:**
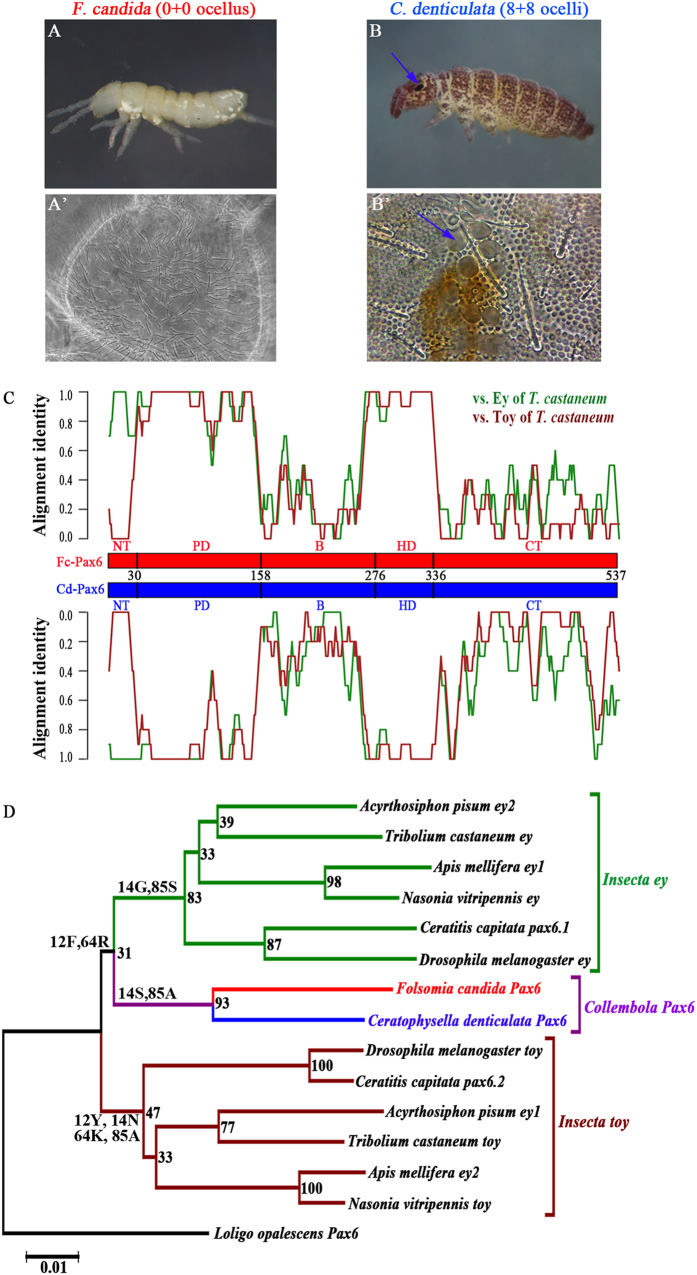
Collembolan Pax6 is more similar to insect Ey than to insect Toy. (**A**–**B**’) Pictures of studied collembolans (with permission granted by the photographer, Dr. Feng Zhang). (**A**) The eyeless *F. candida*. (**A**’) The head of *F.candida*, dorsal view. (**B**) The eyed *C. denticulata* with the eye fields indicated by the blue arrows. (**B**’) Eight ocelli on the left side of the head of *C. denticulata*. (**C**) The heatmap shows percent identity over the Fc-Pax6 and Cd-Pax6 amino acid sequence compared with Ey and Toy from the *T. castaneum*. The red rod and the blue rod demonstrate amino acid sequences of Fc-Pax6 and Cd-Pax6, respectively. The line in green shows comparisons using *T. castaneum* Ey as a query sequence, and the line in brown shows comparisons using *T. castaneum* Toy as the query. The N-terminus (NT), Paired domain (PD), Linker region **B**, Homeodomain (HD), and C-terminus (CT) of Fc-Pax6 and Cd-Pax6 are labeled on the rods, with alignment sites of amino acid sequences. (**D**) The phylogenetic tree using neighbor-joining method for hexapod *Pax6* homologs, based on first and second codon positions of PD and HD. Bootstrap values are indicated at the nodes. Clade-specific significant amino acids of PD (positions 12, 14, 64 and 85) are marked on the corresponding branches.

By PCR amplifications, we detected one *Pax6* gene in the eyeless collembolan *F. candida* (Fig. 1A) and in the eyed collembolan species *C. denticulata* (Fig. 1B), separately. The *Pax6* of *F. candida* (*Fc-Pax6*) is composed of 1,461 nucleotides (that encode 486 amino acids), and the *Pax6* of *C. denticulata* (*Cd-Pax6*) contains 1,422 nucleotides (that encode 473 residues). Five putative regions–NT, PD, B, HD, and CT–of each Pax6 were defined as the 1–8, 9–136, 137–251, 252–311, 312–486 amino acids in Fc-Pax6, and the 1–4, 5–132, 133–243, 244–303, 304–473 amino acids in Cd-Pax6. Their PDs and HDs share 94.5% and 98.3% sequence identity, respectively, whereas their NT, B and CT regions exhibit relatively distinct differences (Supplementary Fig. S1).

Amino acid sequences of Fc-Pax6 and Cd-Pax6 were compared with Ey and Toy of insect *T. castaneum*, respectively, and the heatmap shows both collembolan Pax6 are more similar to Ey than to Toy (Fig. 1C, Supplementary Fig. S2).

In order to illuminate the phylogenetic relationship of collembolan *Pax6* with insect *ey* and *toy*, we conducted the phylogenetic analysis using 14 hexapod *Pax6* sequences from two collembolans and six insects, with the *Pax6* of squid *Loligo opalescens* as the outgroup (Supplementary Fig. S3). The phylogenetic tree based on nucleotide sequences for the first and second codon position of PD and HD suggests that insect *ey* and *toy* are two paralogs, and *Fc-Pax6* and *Cd-Pax6* form another independent clade, which is more close to insect *ey*, although the support value is not high (Fig. 1D).

By comparing the aligned amino-acid sequences of PD from hexapod Pax6, we noticed four significant positions: Phe-12 and Arg-64 are conserved in collembolan Pax6 and insect Ey, but Tyr-12 and Lys-64 are unique in insect Toy. On the contrary, Ala-85 is conserved in collembolan Pax6 and insect Toy, but Ser-85 is peculiar in insect Ey. In addition, for site 14 on the alignment, Gly, Asn and Ser are clade-specific for insect Ey, insect Toy and collembolan Pax6, respectively (Fig. 1D, Supplementary Fig. S4). These positions are supposed to be crucial for the DNA-binding properties^10^,^35^, and therefore, their amino acid substitutions could lead to functional divergence among Ey, Toy and collembolan Pax6.

### Fc-Pax6 exhibits weaker transactivation ability than Cd-Pax6

Pax6 proteins from various animals possess the transactivation ability to induce ectopic *Drosophila*-type compound eyes by means of the *Drosophila* GAL4/UAS system^26^. Using two GAL4 drivers (dpp-GAL4 and ap-GAL4)^36^, we separately misexpressed Fc-Pax6 and Cd-Pax6 to examine their transactivation ability.

**Figure 2:**
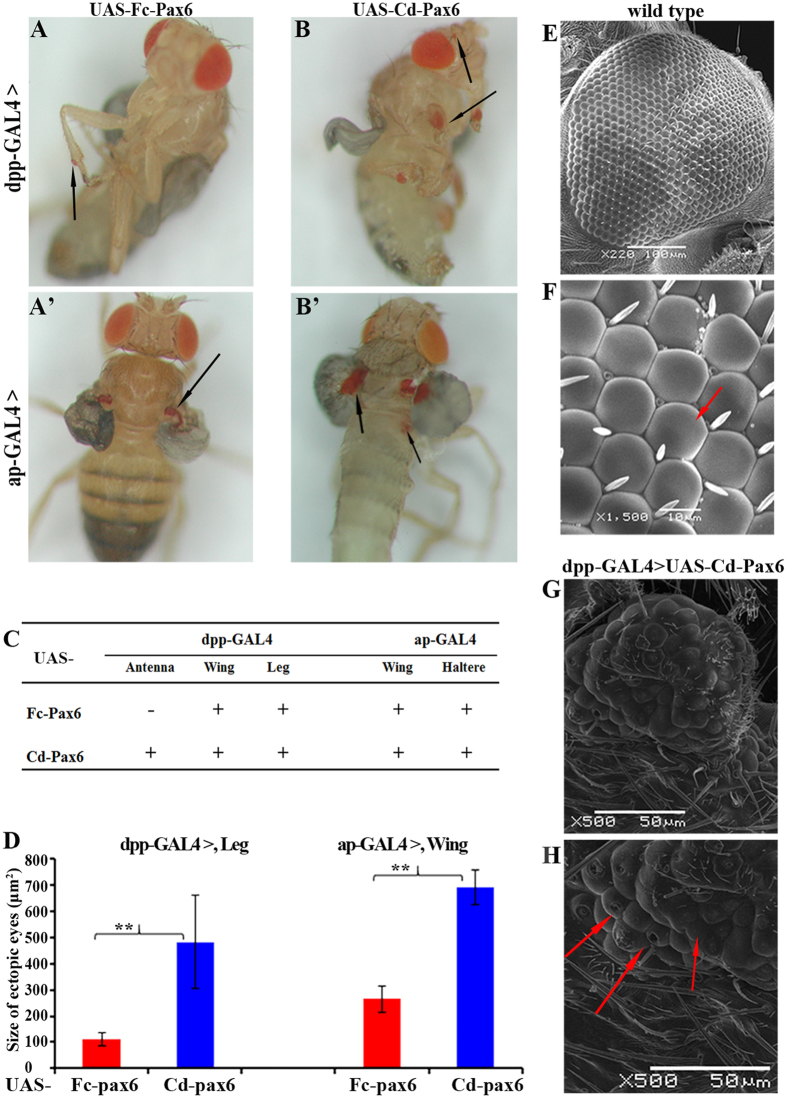
Fc-Pax6 exhibits weaker transactivation ability than Cd-Pax6. (**A**–**B**’) Ectopic *Drosophila-*type eyes induced by intact Fc-Pax6 and Cd-Pax6 driven by dpp-GAL4 and ap-GAL4. Black arrows indicate the positions of ectopic eyes. (**A**) dpp-GAL4 > UAS-Fc-Pax6 (**A**’) ap-GAL4 > UAS-Fc-Pax6. (**B**) dpp-GAL4 > UAS-Cd-Pax6 (**B**’) ap-GAL4 > UAS-Cd-Pax6. (**C**) Comparison of the positions of ectopic eyes induced by Fc-Pax6 and Cd-Pax6 (“+” indicates that ectopic eyes exist and “−” indicates a lack of ectopic eyes). (**D**) Comparison of the sizes of ectopic eyes (** indicates significant differences). (**E**–**H**) SEM images of compound eyes of wild-type *Drosophila* (**E**,**F**) and ectopic eyes on dpp-GAL4 > UAS-Cd-Pax6 (**G**,**H**) (red arrows indicate unusual caves or missing bristle on ommatidia).

Hybrid flies showed four phenotypes: normal wild-type adults, abnormal adults with ectopic eyes or other abnormal phenotypes (i.e., leg, wing, or haltere defects), individuals of incomplete eclosion (pupae or adults stuck on the chrysalis shell), and dead pupae. Both Fc-Pax6 and Cd-Pax6 induced ectopic eyes on the legs and wings via the dpp-GAL4 driver (Fig. 2A–C), and on the wings and haltere via the ap-GAL4 driver (Fig. 2A’–B’). However, antenna-located ectopic eyes were uniquely generated by Cd-Pax6 with the dpp-GAL4 driver (Fig. 2B,C). Moreover, the ectopic eyes induced by Fc-Pax6 were distinctly smaller than those induced by Cd-Pax6 (Fig. 2D). In addition, higher eclosion rates in the pupa stage, along with less incomplete eclosions, were also induced by Fc-Pax6 than by Cd-Pax6, which suggested that Fc-Pax6 produced less effect on *Drosophila* than Cd-Pax6 did (Supplementary Fig. S5A,A’). That is, Fc-Pax6 shows weaker transactivation ability than Cd-Pax6.

In addition, for both Fc-Pax6 and Cd-Pax6, ap-GAL4 resulted in bigger ectopic eyes and more incomplete eclosions than dpp-GAL4 did (Fig. 2D and Supplementary Fig. S5A,A’), in agreement with the previous report that ap-GAL4 is a stronger GAL4 driver than dpp-GAL4^36^.

By using SEM, we then carefully examined the ectopic *Drosophila*-type compound eyes, i.e., those on the legs of dpp-GAL4 > UAS-Cd-Pax6. Compared with the normal *Drosophila* compound eyes, irregularly shaped ectopic eyes are patterned by randomly arranged ommatidium without typical hexagonal facets, and bristles, which normally locate between two ommatidia, are sometimes absent or incorrectly laying on the surface of ommatidia (Fig. 2E–H). The ectopic eyes in *Drosophila* induced by Fc-Pax6 and Cd-Pax6 are very similar to those induced by *Pax6* genes of mouse^3^, *Drosophila*^3^, sea squirt^24^ and squid^25^ in *Drosophila*, which further suggests the conserved function of Pax6, and indicates the key role of Pax6 in collembolan eye development.

### The C-terminus of collembolan Pax6 is indispensable for its transactivation ability

**Figure 3:**
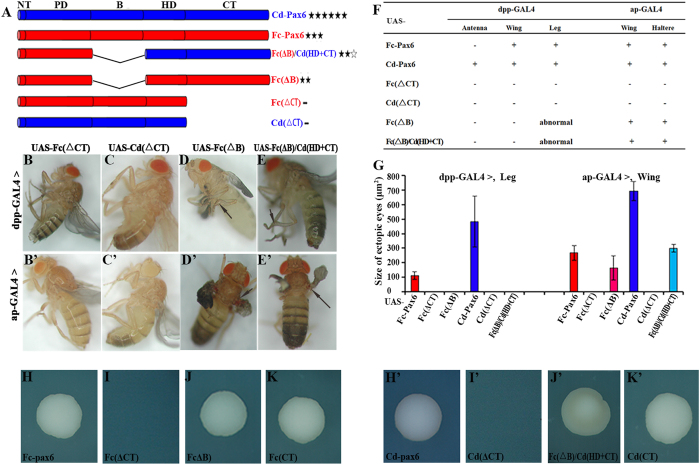
The C-terminus of collembolan Pax6 is indispensable for its transactivation ability. (**A**) Schematic summaries of the original, partially deleted and chimeric Fc-Pax6 and Cd-Pax6 constructs, corresponding to different strengths of transactivation indicated by “★” (“⋆” denotes half strength of the “★”, and “−” denotes a loss of transactivation ability). (**B**–**E**’) Phenotypes of transgenic *Drosophila* carrying the CT or B deletion constructs driven by dpp-GAL4 (**B**–**E**) and ap-GAL4 (**B**’–**E**’). Arrows indicate positions of ectopic eyes. (**F**) Comparison of the positions of ectopic eyes on transgenic *Drosophila* induced by the CT or B deletion constructs (“+” indicates that ectopic eyes exist, and “−” indicates a lack of ectopic eyes). (**G**) Comparison of the sizes of ectopic eyes. (**H**–**K**’) Yeast one-hybrid assay for examining the transactivation domain.

In order to clarify the transactivation domain of collembolan Pax6, deletion constructs were generated by removing the C-termini (CT) or the linker region (B) as Fc (ΔCT), Cd (ΔCT), and Fc(ΔB) (Fig. 3A). We failed to obtain Cd(ΔB); thus, we used chimeric Fc(ΔB)/Cd(HD + CT) as an alternative to Cd(ΔB) (Fig. 3A).

Driven by dpp-GAL4 or ap-GAL4, neither Fc(ΔCT) nor Cd(ΔCT) induced ectopic eyes, and all offspring developed normally into adults in the same manner as the wild type (Fig. 3B–C,B’–C’). Fc(ΔB) and Fc(ΔB)/Cd(HD + C) induced aberrant legs with abnormally curved tibia and shortened tarsus driven by dpp-GAL4 (Fig. 3D–E,F), and induced ectopic eyes driven by ap-GAL4 (Fig. 3D’–E’), although the ectopic eyes were smaller than those induced by the intact *Fc-Pax6* and *Cd-Pax6*, respectively (Fig. 3G).

To further detect whether the C-terminus is the transactivation domain, we conducted the yeast one-hybrid assay. The (-Trp-His-Ade) deficient yeast (A109) could normally grow in (-Trp/-His/-Ade) deficient medium under the control of an activator ligated pGBKT7 vector, which can form a functional GAL4 complex to bind to the UAS sites of the yeast chromosomes, and so the Trp, His and Ade biosynthesis can be initiated. In our study, the deficient yeast strain (AH109) with Fc-Pax6, Cd-Pax6, Fc(CT), Cd(CT), Fc(ΔB), or Fc(ΔB)/Cd(HD + C), can grow normally in deficient culture medium (-Trp/-His/-Ade), whereas transformants with Fc(ΔCT) or Cd(ΔCT) failed to grow in this medium (Fig. 3H–K,H’–K’), which suggested that Fc(ΔCT) or Cd(ΔCT) have no transactivation ability. The transgenic *Drosophila* experiments and the yeast one-hybrid assay together demonstrate that the C-terminus of collembolan Pax6 is indispensable for its transactivation ability.

### The intact C-terminus rather than a small motif is essential for collembolan Pax6 function

**Figure 4:**
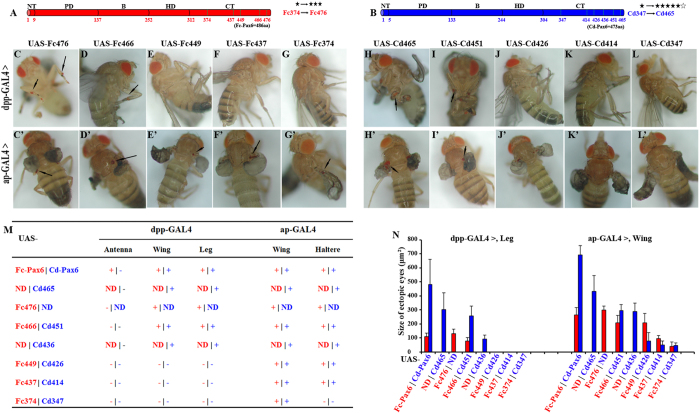
The intact C-terminus rather than a small motif is essential for collembolan Pax6 function. (**A**,**B**) Diagram of constructs with gradual CT deletions (numbers indicate the amino acid positions of Fc-Pax6 (red) and Cd-Pax6 (blue)), corresponding to different strengths of transactivation indicated by “★” (“⋆” denotes half strength of the “★”). (**C**–**L**’) Phenotypes of transgenic *Drosophila* induced by deletion constructs with different CT lengths of Fc-Pax6 and Cd-Pax6 driven by dpp-GAL4 (**C**–**L**) and ap-GAL4 (**C**’–**L**’). Arrows indicate ectopic eyes. (**M**) Comparison of the positions of ectopic eyes on transgenic *Drosophila* induced by deletion constructs with different CT lengths. The construct names connected by “|” indicate the same alignment positions. “ND” indicates that there is no corresponding construct. For the position, “+” indicates that ectopic eyes exist, “−” indicates a lack of ectopic eyes, and “ND” implies no data. (**N**) Comparison of the sizes of ectopic eyes.

The amino acids of C-terminus have been demonstrated essential for vertebrate Pax6 function^21^,^37^,^38^. For collembolan Pax6, we explored the induction assays with the constructs of gradual deletions from the C-termini of Fc-Pax6 and Cd-Pax6, respectively, to check the change of their transactivation ability. The constructs were made as Fc476 (residues 1–476), Cd465 (residues 1–465), Cd436 (residues 1–436), and at the corresponding alignment positions of two proteins: Fc466 (residues 1–466) versus Cd451 (residues 1–451), Fc449 (residues 1–449) versus Cd426 (residues 1–426), Fc437 (residues 1–437) versus Cd414 (residues 1–414), and Fc374 (residues 1–374) versus Cd347 (residues 1–347) (Fig. 4A,B and Supplementary Fig. S1).

Driven by dpp-GAL4, Cd465, Fc476, Fc466|Cd451 and Cd436 induced ectopic eyes, but Fc449|Cd426, Fc437|Cd414, Fc374|Cd347 did not (Fig. 4C–L,M). However, driven by ap-GAL4, all partial CT deletion constructs induced formation of ectopic eyes on the wings (Fig. 4C’-L’). In addition, no deletion constructs induced ectopic eyes on the antenna of flies as Cd-Pax6 did (Fig. 4M). All tests of transgenic *Drosophila* with gradual deletion constructs demonstrated that the more amino acids were removed from the C-termini of collembolan Pax6, the smaller the ectopic eyes, the lower death rates, and the fewer positives were simultaneously generated (Fig. 4N and Supplementary Fig. S5B–C,B’–C’). That is, gradual deletions from the C-terminus of collembolan Pax6 leads to a gradual loss of transactivation ability, indicating that the intact C-terminus rather than a small motif is essential for collembolan Pax6 function.

### Weaker strength of transactivation ability of Fc-Pax6 than Cd-Pax6 is determined by its C-terminus

**Figure 5:**
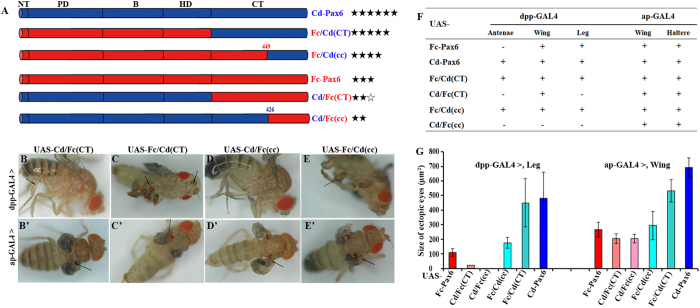
Different transactivation ability between Cd-Pax6 and Fc-Pax6 is determined by their respective C-terminus. (**A**) Schematic summaries of the original Fc-Pax6 (red) and Cd-Pax6 (blue), along with chimeric constructs with whole or partial CT substitutions, corresponding to different strengths of transactivation indicated by “★” (“⋆” denotes half strength of the “★”). (**B**–**E**’) Phenotypes of transgenic *Drosophila* induced by chimeric constructs with CT region substitutions of Fc-Pax6 and Cd-Pax6 driven by dpp-GAL4 (**B**–**E**) and ap-GAL4 (**B**’–**E**’). Arrows indicate positions of ectopic eyes. (**F**) Comparison of the position of ectopic eyes induced by chimeric constructs with CT region substitutions (“+” indicates that ectopic eyes exist, and “−” indicates a lack of ectopic eyes). (**G**) Comparison of the sizes of ectopic eyes.

Furthermore, we constructed chimeric constructs with the C-terminus (CT) or the linker region (B) exchanged, to explore the reason for the different transactivation ability between Fc-Pax6 and Cd-Pax6. Fc/Cd(B) and Cd/Fc(B) were used to exchange the linker region, and Fc/Cd(CT) and Cd/Fc(CT) were used to swap the C-terminus. Fc/Cd(cc) and Cd/Fc(cc) were created by reciprocally exchanging residues 450–486 of Fc-Pax6 with residues 427–473 of Cd-Pax6 (Fig. 5A).

The transactivation ability of Cd/Fc(CT) was similar to that of Fc-Pax6, and the induction ability of Fc/Cd(CT) was similar to that of Cd-Pax6 (Fig. 5B,C,B’). The ability of Fc/Cd(cc) to induce ectopic eyes was obviously stronger than that of Cd/Fc(cc) (Fig. 5D,E,D’). In contrast, ectopic eyes induced by Fc/Cd(B) and Cd/Fc(B) showed similar size, and located at the same locations as those induced by Fc-Pax6 (Supplementary Fig. S6). Therefore, the difference of the transactivation ability between Fc-Pax6 and Cd-Pax6 is determined by their respective C-terminus. The transactivation ability of Fc-Pax6 can be enhanced by replacing its C-terminus with the C-terminus of Cd-Pax6.

## Discussion

Although some studies identified photosensitive organs that develop independently of Pax6, e.g. larval eyes of *Drosophila*^39^,^40^, the recent study of *ey* and *toy* knockdown in *Tribolium* embryo suggested both genes are required for the ocular segment, and the evolution of Pax6-independence of larval eye development in *Drosophila* involved further gene regulatory reorganization from the ancestral direct dependence on Pax6 activity during specification^41^. In this study, for the first time, the *Pax6* coding sequences were cloned from eyeless and eyed collembolan species, which share two highly conserved DNA binding domains (PD and HD) that are found in all other studied animals. Both the eyeless and eyed collembolan species have the full-length *Pax6* cDNA, and both genes have the capacity to induce ectopic eyes on transgenic *Drosophila*. It was further confirmed that the highly conserved *Pax6* is a universal master control gene for eye development in the animal kingdom.

Similar to the studied *Pax6* genes in zebrafish^42^, *Drosophila*^36^, quail^43^, mouse^44^,^45^,^46^, and human^21^,^47^,^48^, the non-conserved C-terminus of the collembolan Pax6 is vital for protein transactivation ability. In our study, gradual deletions from the C-terminal end of collembolan Pax6 led to a gradual loss of activity in inducing ectopic eyes on *Drosophila* (Fig. 4), which indicates that the protein structure rather than a small motif is essential for collembolan Pax6 function. In addition, weaker activity with the linker region deletions also indicates the effects of the protein structure change (Fig. 3).

*Drosophila* Ey can induce ectopic eyes on the antenna, wing, leg and haltere via the dpp-GAL4 driver, and on the wing and haltere via the ap-GAL4 driver. In contrast, *Drosophila* Toy can induce ectopic eyes only on the wing and leg via the dpp-GAL4 driver, and only on the wing via the ap-GAL4 driver^36^. The existence of these two genes is effective for collaborative regulation of the formation of compound eyes. In our study, both Fc-Pax6 and Cd-Pax6 did not induce ectopic eyes on the haltere via the dpp-GAL4 driver (Fig. 2A,B,C), but they did induce ectopic eyes on the haltere via the ap-GAL4 driver (Fig. 2A’). It seems that the transactivation ability of the collembolan Pax6 is between *Drosophila* Ey and Toy, consistent with the intermediate phylogenetic location of collembolan Pax6 between insect Ey and insect Toy (Fig. 1D). On the other hand, the sequence comparison indicated that collembolan Pax6 is more similar to Ey than to Toy (Fig. 1C). As an ancient group of hexapods, Collembola branched off onto its own evolutionary path very early in the evolutionary line to Insecta. Czerny *et al*. proposed that a duplication in *Pax6* gene occurred during insect evolution^10^. However, two *Pax6* genes have also been found in early arthropods: crustacean^49^ and myriapod^50^, which suggests that collembolans probably also have more than one *Pax6* gene, but we failed to obtain its paralog by PCR amplification with different primer pairs and by searching in the transcriptome data of collembolans (unpublished data from our lab).

Numerous and various collembolans are widely distributed in almost all habitats on the earth: in soil, in leaf litter, in moss, under stones, on the surfaces of bark, on mushrooms and flowers, in caves, in ant and termite nests, in the intertidal zones of coasts, on the surfaces of water, and in snow fields. They even have been found in snow in Antactica and on the highest peaks of the Himalayas^29^. Generally speaking, most collembolan species with eyes are commonly found on the surface of fallen leaves, litter, moss, or water, while most eyeless species usually live in soils or caves and have slightly colored bodies or no pigmentation. Different collembolan species in the same genus usually possess a variable number of ocelli. Morphologists believe that the common ancestor of collembolans had eyes (at least 6 + 6 ocelli, maybe one or two more)^51^,^52^. This hypothesis is supported by the evidence that the oldest Devonian fossil collembolan, *Rhyniella praecursor* (it lived 400 million years ago and is among the oldest known records of terrestrial animals), had 8 + 8 ocelli^53^. Our molecular data further support the mono-origin of collembolan eyes, since Pax6 from the eyeless and eyed collembolans have significant sequence similarity and functional conservation in generating ectopic eyes.

For *F. candida* with no external eye on the head surface, although the image-resolving and color vision capacity was lost, translucent interocular vesicle-containing subcutaneous rhabdomes (non-ocular photoreceptors) still allow them to detect and distinguish UV, white light and darkness^34^,^54^. In our study, the Pax6 of *F. candida* retained the transactivation ability to induce the formation of ectopic eyes on *Drosophila*, but its activity was obviously weaker than that of eyed *C. denticulata* (Fig. 2). One of the reasonable explanations is that the *Fc-Pax6* gene might have accumulated more mutations, particularly at some key functional sites of C-terminus of Fc-Pax6 (Figs 3, 4, 5), most likely because the eyes are not important for *F. candida* living in soil and thus Fc-Pax6 is under lower selection pressure compared with Cd-Pax6. Eye degeneration is a good energy efficiency strategy for the evolution of soil-dwelling collembolans.

The morphological and physiological adaptations to diverse habitats are easily observed, but the underlying genetic mechanisms are difficult to elucidate. Our study provides the first molecular evidence to support the degeneration of collembolan eyes rather than an absence of eyes in the collembolan ancestor. This is a good case of adaptive evolution of eye regression. In the future, studies on *Pax6* from more collembolan species, as well as more genes involved in the collembolan eye development will help us better understand the genetic mechanism responsible for the evolution of collembolan eyes.

## Materials and Methods

### Materials

The eyeless collembolan species, *F. candida* Willem, 1902 (Entomobryomorpha: Isotomidae), was kindly provided by Aarhus University, Denmark. The eyed species, *C. denticulata* (Bagnall, 1941) (Poduromorpha: Hypogastruridae) with 8 + 8 ocelli, was collected in Shanghai, China. Both species were cultured at 21 °C and 75% humidity in the laboratory for a long period and were fed with granulated dry yeast.

### Collembolan *Pax6* cDNA cloning

Genomic DNA of adult collembolans was extracted using the Wizard® SV Genomic DNA Purification System (Promega). Partial PD fragments were amplified using genomic DNA as template, followed by the nested PCR strategy of Arendt *et al*.^55^ with two sets of degenerate primers (Supplementary Table S1A).

Total RNA was isolated from mixed individuals at different developmental stages (embryos, larvae and adults) of each collembolan species using TRIzol reagent (Qiagen), and cDNA was synthesized using SuperScript™ III Reverse Transcriptase (Invitrogen). Gene-specific primers (Supplementary Table S1A) were designed based on the obtained *Pax6* sequences of each species, and 5′ and 3′ RACE with the cDNA templates were performed according to the instructions in the 5′ full RACE kit and 3′ full RACE kit from Takara Bio, Inc. (Dalian, China). PCR products were cloned into the PMD19-T vector (TaKaRa, D6013), and then transformed into Top10 competent cell (Tiangen, CB104–03). Positive clones were sequenced with M13–47 and M13–48 primers by the commercial company Sangon (Shanghai, China). All sequencing reads were assembled with the program SeqMan in the DNASTAR package^56^.

### Comparison of sequence similarity and phylogenetic analysis

With reference to the corresponding domain of Pax6 of studied animals^6^,^7^,^8^,^9^,^10^,^11^,^12^,^13^,^14^,^15^, the amino acid sequences of Fc-Pax6 and Cd-Pax6 were aligned with MEGA6^57^ and five putative regions (NT, PD, B, HD, and CT) were defined, respectively^5^ (Fig. 1C and Supplementary Fig. S1).

Using the amino acid sequences of Ey and Toy from the insect *T. castaneum* as the query, a heatmap was designed to compare the sequence similarity of both collembolan Pax6 to insect Ey and Toy, respectively (Supplementary Fig. S2). Four amino acid sequences were aligned using MEGA6^57^. Similarity was calculated for every 10-bp window using a custom Perl script and heatmap was plotted using R.

12 insect *Pax6* (including 6 *ey* and 6 *toy*) and one squid *Pax6* were retrieved from the NCBI. Together with the two collembolan *Pax6* we obtained, the nucleotide sequences of conserved PD and HD of 15 Pax6 homologs were aligned using MEGA6^57^. Gene details are presented in Supplementary Table S2, and the multiple sequence alignment is presented in Supplementary Fig. S3. With the squid *Pax6* sequence as the outgroup, a neighbor-joining (NJ) tree was conducted with MEGA6^57^ using the p-distance model and 1000 bootstrap replicates based on the first and the second codon sites of *Pax6* nucleotide sequences of PD and HD.

### Manipulation of collembolan *Pax6*

Deletion and chimeric constructs were generated via standard recombinant PCR techniques using high-fidelity PCR Polymerase (Takara). All constructs were confirmed by sequencing, and all primers used in constructing primitive and modified *Pax6* genes are listed in Supplementary Table S1B. The PCR conditions and cloning strategies for each construct are available upon request.

First, the full-length *Pax6* coding sequence of *F. candida* (1461 nucleotides that encode 486 amino acids) was amplified using primers with *Not*I and *Xho*I restriction sites, and the whole *Pax6* coding sequence of *C. denticulata* (1422 nucleotides that encode 473 amino acids) was amplified using primers with *Kpn*I and *Xba*I restriction sites. Then, the individual domains were amplified from plasmids that contained the intact *Pax6* open reading frames, separately.

The unique restriction enzyme *Nde*I site was added to the end of PD and the starting point of HD to facilitate the creation of constructs with the linker region removed. The constructs of gradual deletions according to the alignment of the C-termini of Fc-Pax6 and Cd-Pax6 were built with reverse primers to specific positions. Primers with overlapping sequences or amplified fragments with overlaps were used to join neighboring segments to create chimeric genes. Diagrams of each construct are presented in Figs 3A,4A and 5A, as well as in Supplementary Fig. S6A.

### Transgenic *Drosophila* and phenotype analysis

Each gene fragment was excised by its corresponding restriction endonuclease, and then subcloned into a pUAST-attB vector. Extracted plasmid (>75 μg) (Plasmid Midi Kit, QIAGEN) for each construct was injected into *Drosophila* embryos at the 2^−^cell stage at the Core Facility of Drosophila Resource and Technique (SIBS, CAS). After rearing for 11 days at 25 °C, adult *Drosophila* with red eyes were selected as positive transformers for the line balance, and then for generating homozygotes of each *Drosophila* line.

Two GAL4 stocks, ap-GAL4 (BS3041) and dpp-GAL4 (BS1553), were purchased from the Bloomington Fly Stocks. Homozygous male transgenic *Drosophila* of each gene construct and virgin GAL4 drivers (dpp-GAL4 and ap-GAL4) were selected and raised together for 24 hours, and then all adults were removed. Hybrid embryos at nearly the same development stage were kept in the culture for 5–6 days at 25 °C.

Hybrid flies with ectopic eyes or other abnormal phenotypes (i.e., leg, wing, or haltere defects) were defined as positives. Ectopic eyes were observed under a light microscope (Nikon 600) and a SEM (JEOL JSM-6360LV). For size comparisons, ectopic eyes on legs (crossing with dpp-GAL4 driver) or wings (crossing with ap-GAL4 driver) were treated as a rectangle to calculate their areas. The average sizes of 20 ectopic eyes on different organisms of each line were calculated for comparing the transactivation strength of each construct.

Three replicates of 100 individuals of the 3rd instar larvae (5–6 days) of each line were shifted into new dishes for the purpose of counting the dead pupae, positive pupae, and positive adults. The eclosion rate (adults/100), positive rate ((incomplete eclosions + abnormal adults)/(100-dead pupae)), and rate of incomplete eclosions in all positives (incomplete eclosions/(incomplete eclosions + abnormal adults)) were calculated separately to further compare the transactivation ability of each transgene in the flies.

### Yeast one-hybrid assay

The pGBKT7 vector that has an open reading frame for Trp biosynthesis inframes with the GAL4 DNA binding domain (GAL4-DBD) and a (-Trp-His-Ade) deficient yeast strain AH109 were used to test transcriptional activation (Yeast Protocols Handbook, Clontech Laboratories, Inc.)^36^. *Fc-Pax6, Cd-Pax6, Fc(*Δ*CT), Cd(*Δ*CT), Fc(CT)*, and *Cd(CT)* were amplified by the primers with endonuclease sites from the pUAST-attB vector (the primers are listed in Supplementary Table S1C). Each excised fragment was ligated with DBD vectors, and then transformed into AH109 yeast strain. All transformants were plated on Trp-deficient (-Trp) solid medium for two days at 30 °C. Positive clones were selected and diluted in the deficient liquid culture medium (-Trp/-His/-Ade), which was made by adding 20 × Leu into 10 × Dropout/-Trp/-His/-Ade/-Leu (Clontech), and were then plated on the deficient solid culture medium (-Trp/-His/-Ade) to observe the growth of different yeast lines. Each assay was replicated three times.

## Additional Information

**How to cite this article**: Hou, Y.-N. *et al. Pax6* in Collembola: Adaptive Evolution of Eye Regression. *Sci. Rep.* **6**, 20800; doi: 10.1038/srep20800 (2016).

## Supplementary Material

Supplementary Information
