# Supplementary material for: Pax6 in Collembola: Adaptive Evolution of Eye Regression
Source: Sci Rep. 2016 Feb 9;6:20800. doi: 10.1038/srep20800 (PMC4746759; doi:10.1038/srep20800)
Supplement: Supplementary Information [file srep20800-s1.pdf]

Supplementary Information

Pax6 in Collembola: Adaptive Evolution of Eye Regression

Ya-Nan Hou, Sheng Li*, Yun-Xia Luan*

Key Laboratory of Insect Developmental and Evolutionary Biology, Institute of Plant Physiology & Ecology, Shanghai Institutes for Biological Sciences, Chinese Academy of Sciences, Shanghai, China

*Corresponding authors: E-mail: lisheng01@sibs.ac.cn; yxluan@sibs.ac.cn

Table S1. All primers used in the study.

Table S1A. Primers used for the whole collembolan *Pax6* cDNA amplification.

Primer name	Sequences (5’-3’)
**Partial Paired-domain**	
P-Pax6-F	GGICAYWSIGGNGTIAAYCA
P-Pax6-R	GGIGCIMGICCITGYGAYAT
P-Pax6-nF	ARNARNCKRTCNCKDATYTCCCA
P-Pax6-nR	TCNCKDATYTCCCANGGRAA
**5’ and 3’ Race**	
Fc-Pax6-5’-outer	TATCGACGGACATTCTCTCTTGTAATTG
Fc-Pax6-5’-inner	ATCCAGTTTCATAGTACCGTCCCA
Fc-Pax6-3’-outer	CCAATGGCTGTGTCTCCAAAATCT
Fc-Pax6-3’-inner	GTGAACAAAATAGCCAATTACAAG
Cd-Pax6-5’-outer	GATTGACGGACATTCTCGCTTGTA
Cd-Pax6-5’-inner	CCTCCGATGGCTCGCGGTCGAAT
Cd-Pax6-3'-outer	TGTGTTTCCAAAATACTCGGCAGG
Cd-Pax6-3'-inner	GTCCACCGGATTGCTCAGTAC
**Full length of collembolan *Pax6* gene**	
Fc-Pax6-for-*Not*I	ATAAGAATGCGGCCGCATGATACACGCAGACAACCAA
Fc-Pax6-rev-*Xho*I	CCGCTCGAGTTAGTTTTTCTCATGGACCGT
Cd-Pax6-for-*Kpn*I	GGGGTACCATGCCGCACAAAGGGCACAG
Cd-Pax6-rev-*Xba*I	GCTCTAGATTACTGAAGTCGAGACCAGTAC

Table S1B. Primers used for deletion and chimeric constructs of collembolan Pax6.

Sites	Primer name	Sequences (5'-3')
**Deletions in C-terminal region of Fc-Pax6**		
Fc-478	Fc-478r-*Xho*I	CCGCTCGAGTTAAAGTGAATGATAAGAAGGCA
Fc-468	Fc-468r-*Xho*I	CCGCTCGAGTTATGGCGCAGCGGAATATTGAGC
Fc-449	Fc-449r-*Xba*I	GCTCTAGAGCTTACGAATAGGTAGAGGCGAGC
Fc-437	Fc-437r-*Xba*I	GCTCTAGATTAAGATCGTGCCATGCATGGATA
Fc- 374	Fc-374r-*Xba*I	GCTCTAGATTAAGGATGGATCGGGTAGACGGA
Fc-311	Fc(ΔCT)-*Xba*I	GCTCTAGATTACTCTTCACGTCTCCATTTTGC
**Deletions in C-terminal region of Cd-Pax6**		
Cd-466	Cd-466r-*Xba*I	TCTAGATTAATCGGAGGAGTGTCCAGGAAC
Cd-458	Cd-458r-*Xba*I	TCTAGATTAGGCTACTGGAACAGACACGCC
Cd-451	Cd-451r-*Xba*I	TCTAGATTAGGGTGAAATAAGTCCTGTTGA
Cd-436	Cd-436r-*Xba*I	TCTAGATTATTGGTACGGATCAGCCGGTCT
Cd-426	Cd-426r-*Xba*I	TCTAGATTAGGAGTATGTCGAAGCGAGCCC
Cd-414	Cd-414r-*Xba*I	TCTAGATTAATGGGCTCGGGTCATGCACGG
Cd-347	Cd-347r-*Xba*I	TCTAGATTAAGATGGGTGAAAGGTGTTTCC
Cd-303	Cd(ΔCT)-*Xba* I	TCTAGATTACTCCTCCCGCCGCCATTTAGC
**Chimeric constructs and linker region deletion constructs**		
whole CT	HD-99-for	GAGCGKACYCAYTACCCAGAC
	HD-201-rev	YTTCTCYTCMCGYCKCCATTT
partial CT	Pax6-(cc)-for	GGCTCGCTTCGACVTAYTCVC
	Pax6-(cc)-rev	GGAGTATGTCGARGCBARHCC
B region	PD-(128aa)-for	AGYGTRTCRTCHATCAAYCGHGTBCTC
	PD-(128aa)-rev	GAGMACKCGRTTRATWGATGATACRCT
	HD-(1aa)-for	ACKTCCTTCACSAAYGARCARATCGAR
	HD-(1aa)-rev	YTCGATYTGYTCRTTSGTGAAGGAMGT

Table S1C. Primers used for the constructs in the yeast one-hybrid assay.

**Fragment**	**Primer name**	**Sequence (5'-3')**
Fc-Pax6	Fc-1f-*Nde*I	GGAATTCCATATGATGATACACGCAGACAACCAA
	Fc-r-*Pst*I	AACTGCAGTTAGTTTTTCTCATGGACCGTTTC
Fc(ΔCT)	Fc-1f-*Nde*I	GGAATTCCATATGATGATACACGCAGACAACCAA
	Fc-hd-r-*Pst*I	AACTGCAGTTACAGTTTCTCTTCACGTCTCCAT
Fc(CT)	Fc-ct-f-*Nde*I	GGAATTCCATATGAAACTGAGAAACCAACGCCGG
	Fc-r-*Pst*I	AACTGCAGTTAGTTTTTCTCATGGACCGTTTC
Fc(ΔB)	Fc-1f-*Nde*I	GGAATTCCATATGATGATACACGCAGACAACCAA
	Fc-r-*Pst*I	AACTGCAGTTAGTTTTTCTCATGGACCGTTTC
Fc(ΔB)/Cd(HD+CT)	Fc-1f-*Nde*I	GGAATTCCATATGATGATACACGCAGACAACCAA
	Cd-r-*Pst*I	AACTGCAGTTACTGAAGTCGAGACCAGTA
Cd-Pax6	Cd-1f-*Nde*I	GGAATTCCATATGATGCCGCACAAAGGGCACAGT
	Cd-r-*Pst*I	AACTGCAGTTACTGAAGTCGAGACCAGTA
Cd(CT)	Cd-ct-f-*Eco*RI	GGAATTCAAGCTAAGAAATCAACGGCGT
	Cd- r-*Pst*I	AACTGCAGTTACTGAAGTCGAGACCAGTA
Cd(ΔCT)	Cd-1f-*Nde*I	GGAATTCCATATGATGCCGCACAAAGGGCACAGT
	Cd-hd-r-*Eco*RI	AACTGCAGTTACTCCTCCCGCCGCCATTTAGC

Table S2. Information on *Pax6* genes used in the phylogeny study.

Taxon			Species	Gene name	GenBank accession No.
**Arthropoda**	**Hexapoda, Collembola**	**Entomobryomorpha, Proisotominae**	***Folsomia candida***	***Folsomia candida Pax6***	**KP765728**
		**Poduromorpha, Hypogastruridae**	***Ceratophysella denticulata***	***Ceratophysella denticulata Pax6***	**KP765727**
	Hexapoda, Insecta	Hemiptera, Aphididae	*Acyrthosiphon pisum*	*Acyrthosiphon pisum ey1*	XM_001944211
				Acyrthosiphon pisum ey2	XM_003246811
		Hymenoptera, Apidae	*Apis mellifera*	*Apis mellifera ey1*	XM_006559854
				Apis mellifera ey2	XM_006565376
		Hymenoptera, Pteromalidae	*Nasonia vitripennis*	*Nasonia vitripennis toy*	XM_001602773
				Nasonia vitripennis ey	XM_001601268
		Coleoptera, Tenebrionidae	*Tribolium castaneum*	*Tribolium castaneum ey*	EU169112
				Tribolium castaneum toy	XM_008193905
		Diptera, Tephritidae	*Ceratitis capitata*	*Ceratitis capitata Pax6.1*	GAMC01001807
				Ceratitis capitata Pax6.2	XM_004537918
		Diptera, Drosophilidae	*Drosophila melanogaster*	*Drosophila melanogaster ey*	NM_001014693
				Drosophila melanogaster toy	NM_079899
**Mollusca**	Cephalopoda	Teuthida, Loliginidae	*Loligo opalescens*	*Loligo opalescens Pax6*	U59830

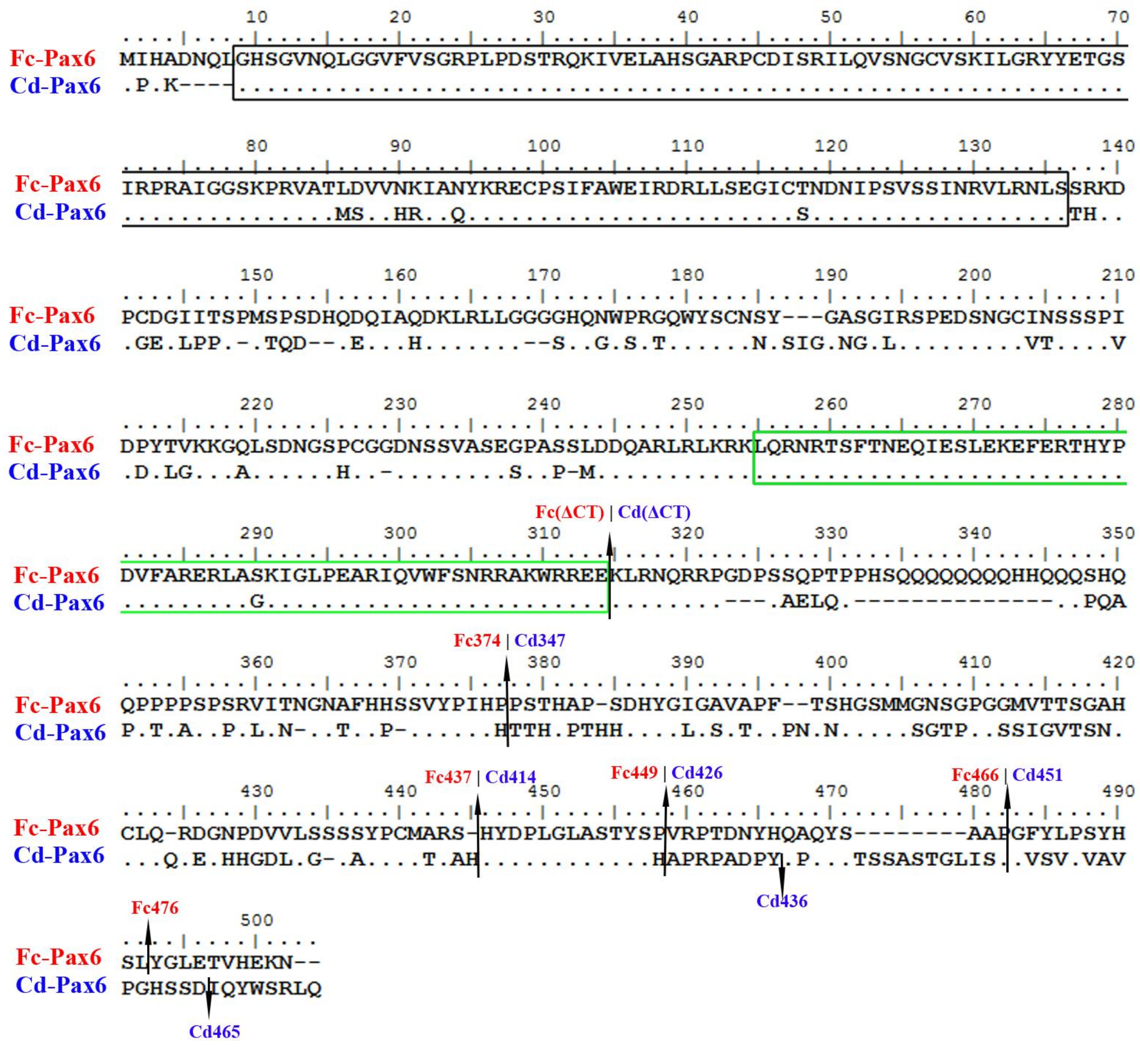

Figure S1. Alignment of the full-length amino-acid sequences of Fc-Pax6 and Cd-Pax6. Black box: Paired domain (PD). Green box: Homeodomain (HD). Black arrows indicate the sites for building deletion constructs of Fc-Pax6 (Red numbers) and the corresponding sites of Cd-Pax6 (Blue numbers).

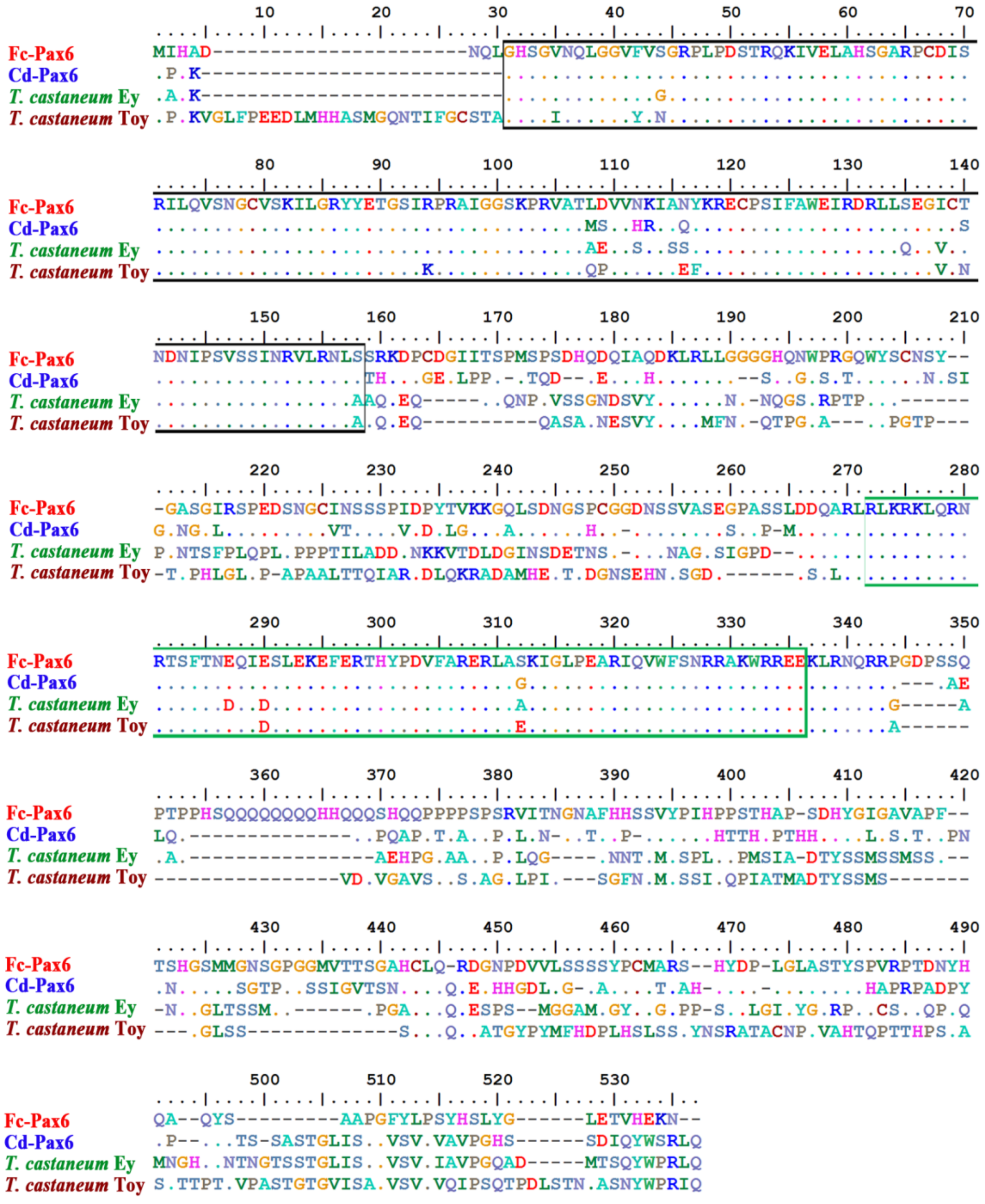

Figure S2. Alignment of the full-length amino-acid sequences of Fc-Pax6, Cd-Pax6, *T. castaneum*-Ey and *T. castaneum*-Toy. Black box: Paired domain (PD). Green box: Homeodomain (HD).

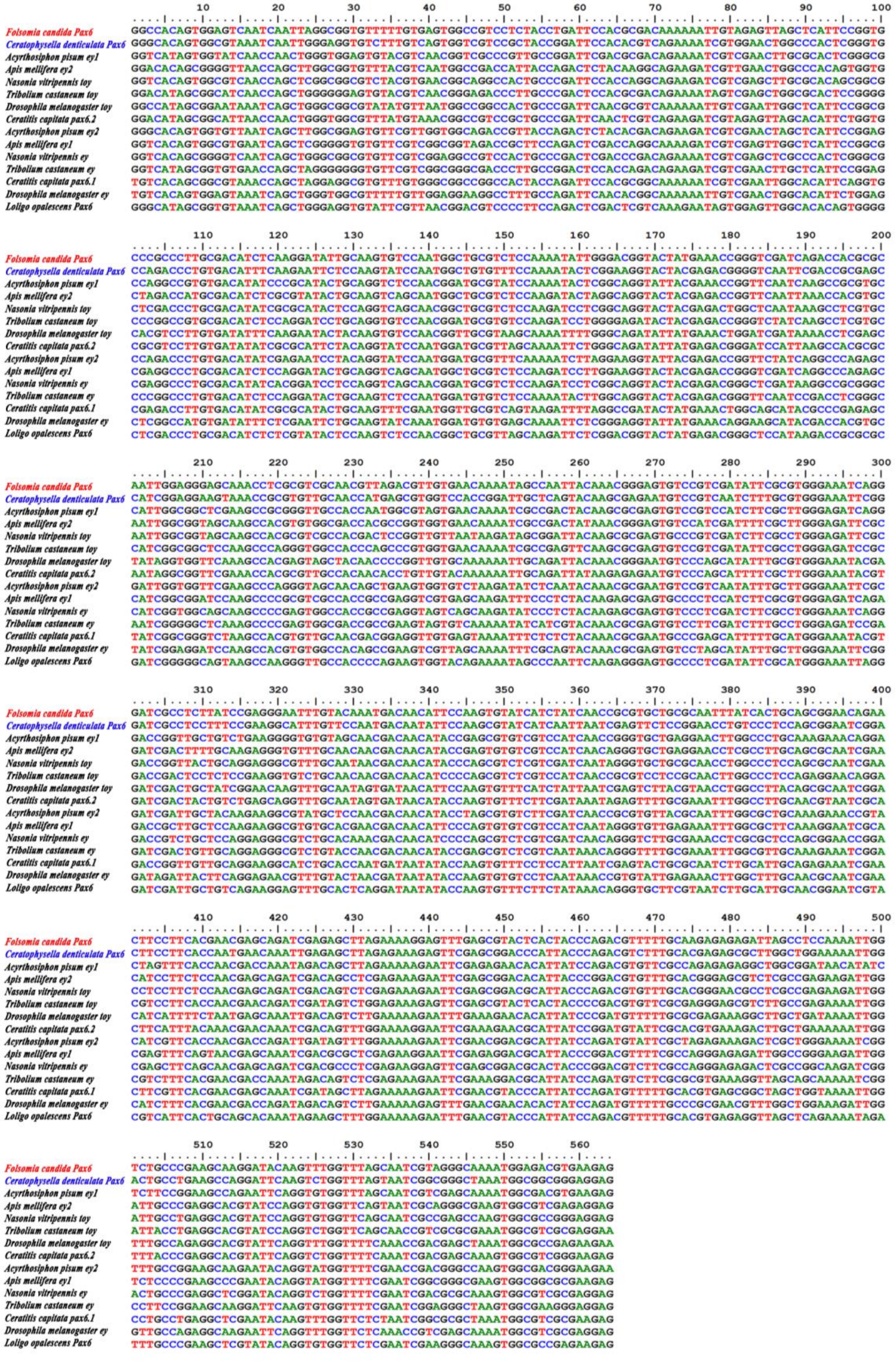

Figure S3. Aligned nucleotide sequences of the PD and HD of 15 *Pax6* genes from 6 insects (six *ey* and six *toy*), 2 collembolans and 1 squid.

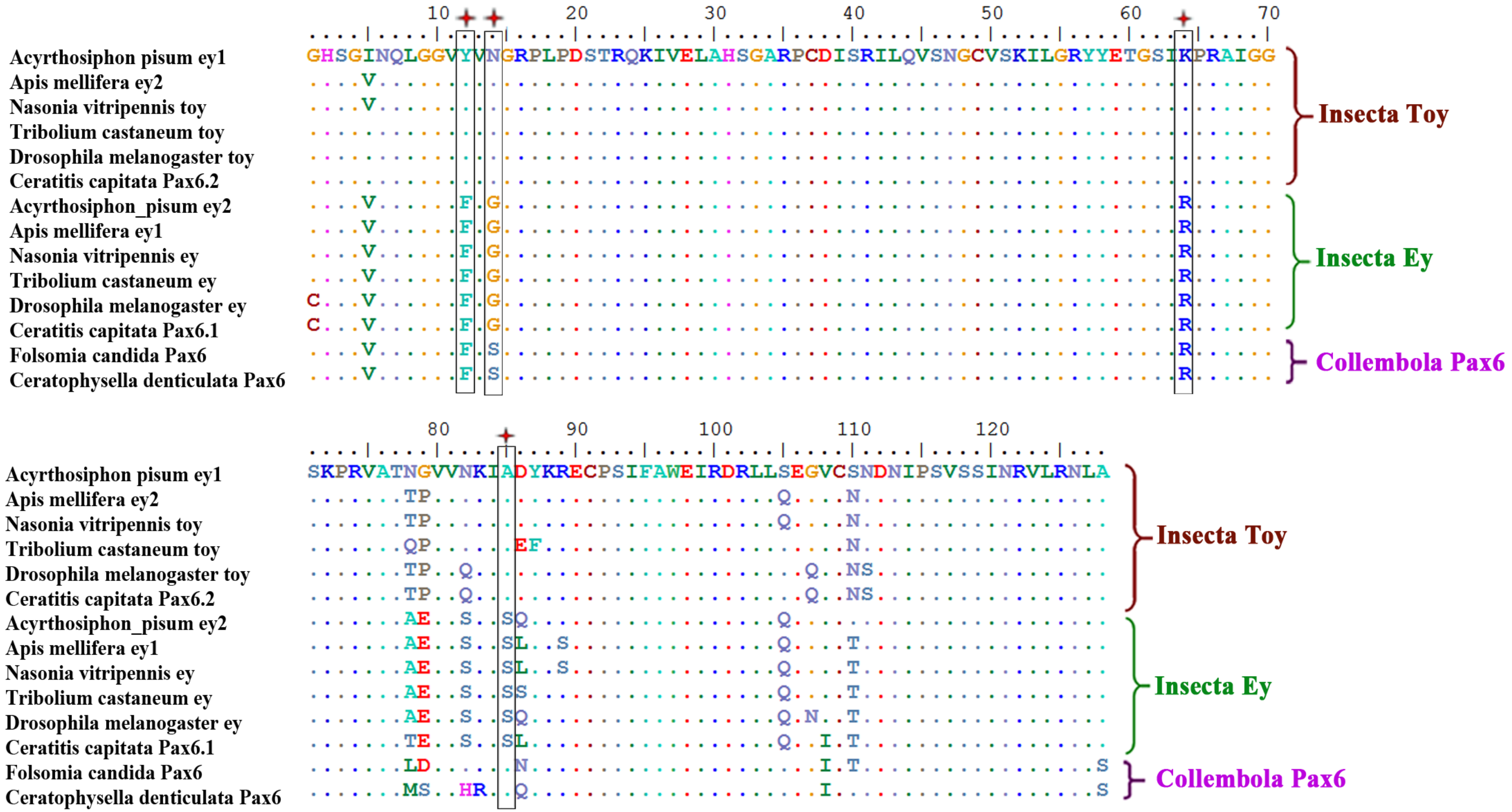

Figure S4. Aligned amino-acid sequence of PD of 14 Pax6 from 6 insects (six Ey and six Toy) and 2 collembolans. Clade-specific significant positions in the amino-acid sequences of PD: 12, 14, 64 and 85 are highlighted in boxes. Three hexapod Pax6 clades are labeled right.

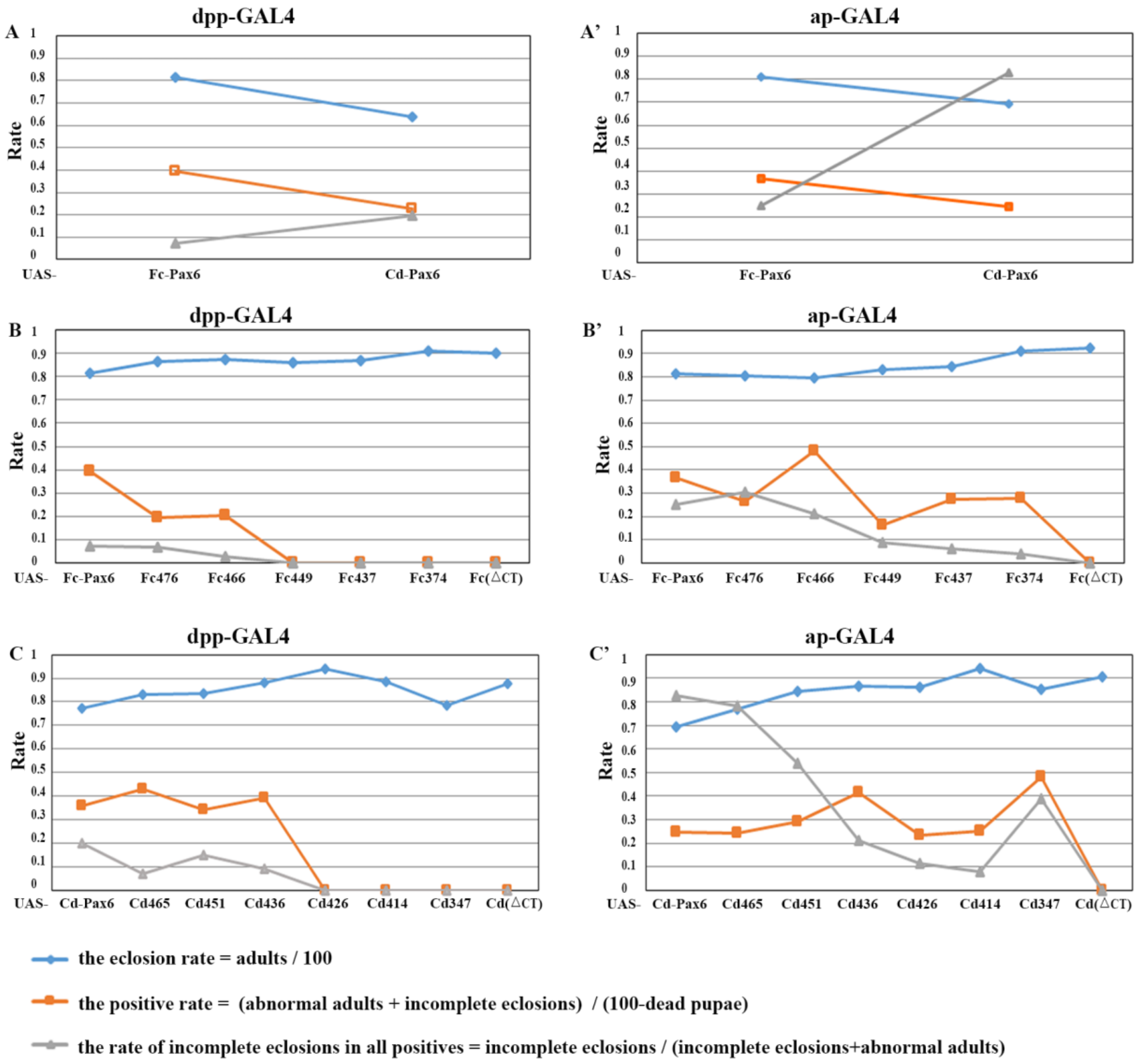

Figure S5. Comparison of the eclosion rates, positive rates, and incomplete eclosion rates in transgenic *Drosophila* induced by Fc-Pax6, Cd-Pax6, or gradual deletion constructs of Fc-Pax6 and Cd-Pax6 driven by dpp-GAL4 (A-C) and ap-GAL4 (A’-C’).

(A, A’) Comparisons between transgenic *Drosophila* carrying Fc-Pax6 and Cd-Pax6.

(B, B') Comparisons among transgenic *Drosophila* carrying Fc-Pax6 constructs with different CT lengths.

(C, C') Comparisons among transgenic *Drosophila* carrying Cd-Pax6 constructs with different CT lengths.

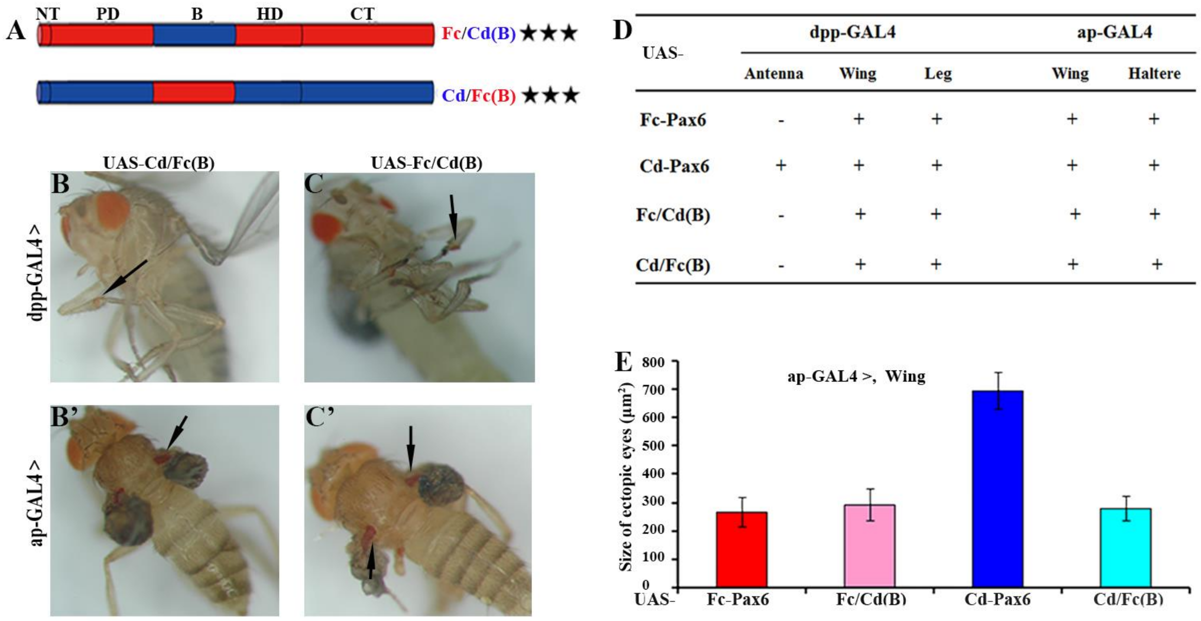

Figure S6. Exchange of the linker region (B) of two collembolan Pax6 proteins showed similar transactivation ability as intact Fc-Pax6.

(A) Diagram of chimeric constructs with B substitution, corresponding to similar strengths of transactivation ability indicated by "★".

(B-C') Phenotypes of transgenic *Drosophila* induced by B substituted constructs of Fc-Pax6 and Cd-Pax6 driven by dpp-GAL4 (B-C) and ap-GAL4 (B'-C'). Arrows indicate ectopic eyes.

(D) Comparison of the position of ectopic eyes induced by B substituted constructs ("+" indicates that ectopic eyes exist, and "-" indicates a lack of ectopic eyes).

(E) Comparison of the sizes of ectopic eyes.
